# Supplementary material for: Parenting a Child with a Neurodevelopmental Disorder during the Early Stage of the COVID-19 Pandemic: Quantitative and Qualitative Cross-Cultural Findings
Source: Int J Environ Res Public Health. 2022 Dec 28;20(1):499. doi: 10.3390/ijerph20010499 (PMC9819011; doi:10.3390/ijerph20010499)
Supplement: Supplementary file 1 [file ijerph-20-00499-s001.zip › ijerph-2039201-supplementary.pdf]

Table S1. Bivariate correlations among the study variables.

	Parental age	Family SES	Child age	ΔTherapy	Covid-19 SDQ_ext	Pre-Covid-19 SDQ_ext	ΔSDQ_ext	Covid-19 PSS	Pre-Covid-19 PSS	ΔPSS	Covid-19 GHQ
Parental age	1.000	0.192*<br>0.022	0.499**<br>0.000	0.112<br>0.186	-0.154<br>0.067	-0.131<br>0.118	-0.067<br>0.428	-0.065<br>0.438	0.006<br>0.940	-0.112<br>0.185	-0.160<br>0.056
Family SES		1.000	0.015<br>0.855	0.026<br>0.755	-0.225**<br>0.007	-0.204*<br>0.015	-0.080<br>0.340	-0.034<br>0.686	-0.101<br>0.232	0.092<br>0.274	-0.075<br>0.372
Child age		 .	1.000	0.254**<br>0.002	0.021<br>0.806	0.126<br>0.133	-0.164<br>0.050	0.011<br>0.895	0.103<br>0.219	-0.132<br>0.116	0.002<br>0.978
ΔTherapy				1.000	-0.144<br>0.088	-0.073<br>0.393	-0.143<br>0.090	-0.096<br>0.258	-0.087<br>0.307	-0.025<br>0.771	-0.184*<br>0.029
Covid-19 SDQ_ext					1.000	0.833**<br>0.000	0.471**<br>0.000	0.417**<br>0.000	0.327**<br>0.000	0.181*<br>0.030	0.311**<br>0.000
Pre-Covid-19 SDQ_ext						1.000	-0.095<br>0.259	0.288**<br>0.000	0.415**<br>0.000	-0.149<br>0.076	0.144<br>0.087

ΔSDQ_ext	1.000	0.291**	-0.074	0.564**	0.331**
		0.000	0.378	0.000	0.000
Covid-19 PSS		1.000	0.783	0.436**	0.438**
			0.000	0.000	0.000
Pre-Covid-19 PSS			1.000	-0.220**	0.218**
				0.008	0.009
ΔPSS				1.000	0.370**
					0.000
Covid-19 GHQ					1.000
